# Supplementary material for: Impact of Dietary Crude Protein Level on Hepatic Lipid Metabolism in Weaned Female Piglets
Source: Animals (Basel). 2021 Jun 18;11(6):1829. doi: 10.3390/ani11061829 (PMC8235084; doi:10.3390/ani11061829)
Supplement: Supplementary file 1 [file animals-11-01829-s001.zip › animals-1191254 sup.pdf]

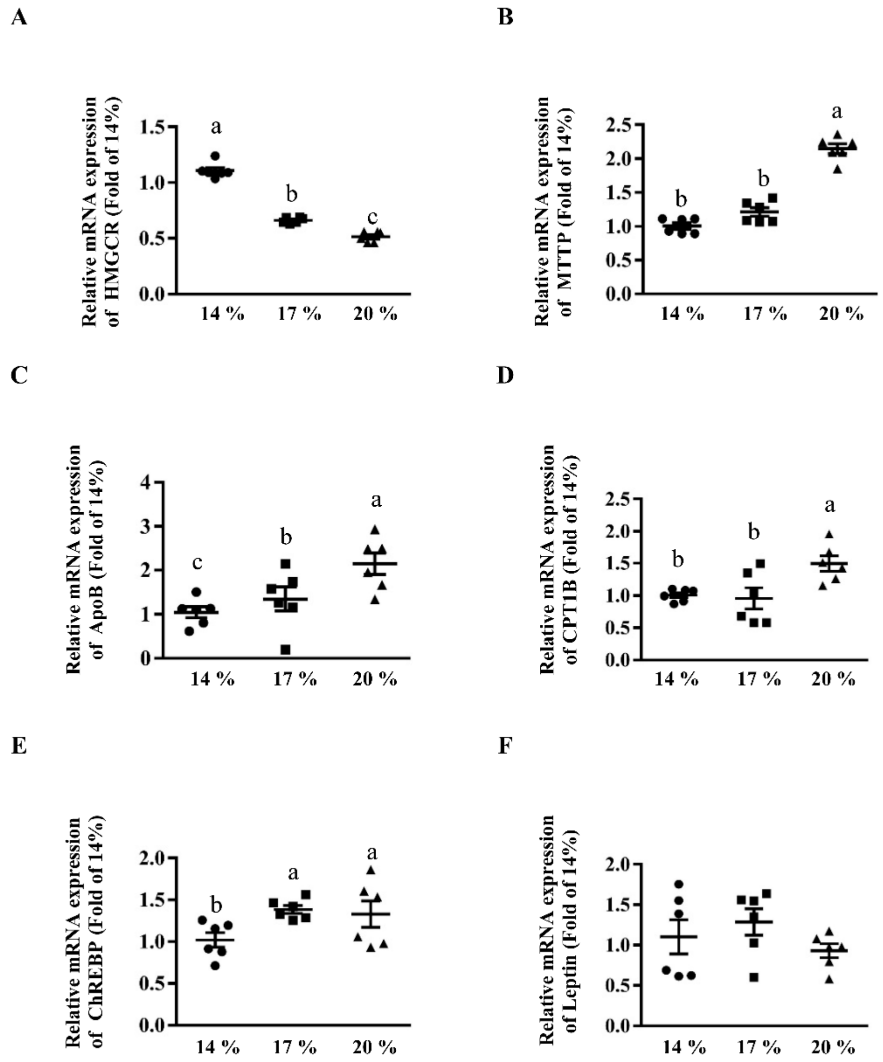

Supplemental Figure S1. Effects of dietary crude protein level on liver genes expression. HMGCR (A), MTTP (B), ApoB (C), CPT1B (D), ChREBP (E), and Leptin (F) mRNA abundance in liver weaned piglets fed different dietary crude protein. Values are means ± SEMs, n = 6. Means without a common letter differ, $P < 0.05$. ApoB, Apolipoprotein B; ChREBP, Carbohydrate responsive element binding protein; CPT1B, Carnitine O-palmitoyltransferase 1; HMGCR, HMG-CoA Reductase; MTTP, Microsomal triglyceride transfer protein large subunit.

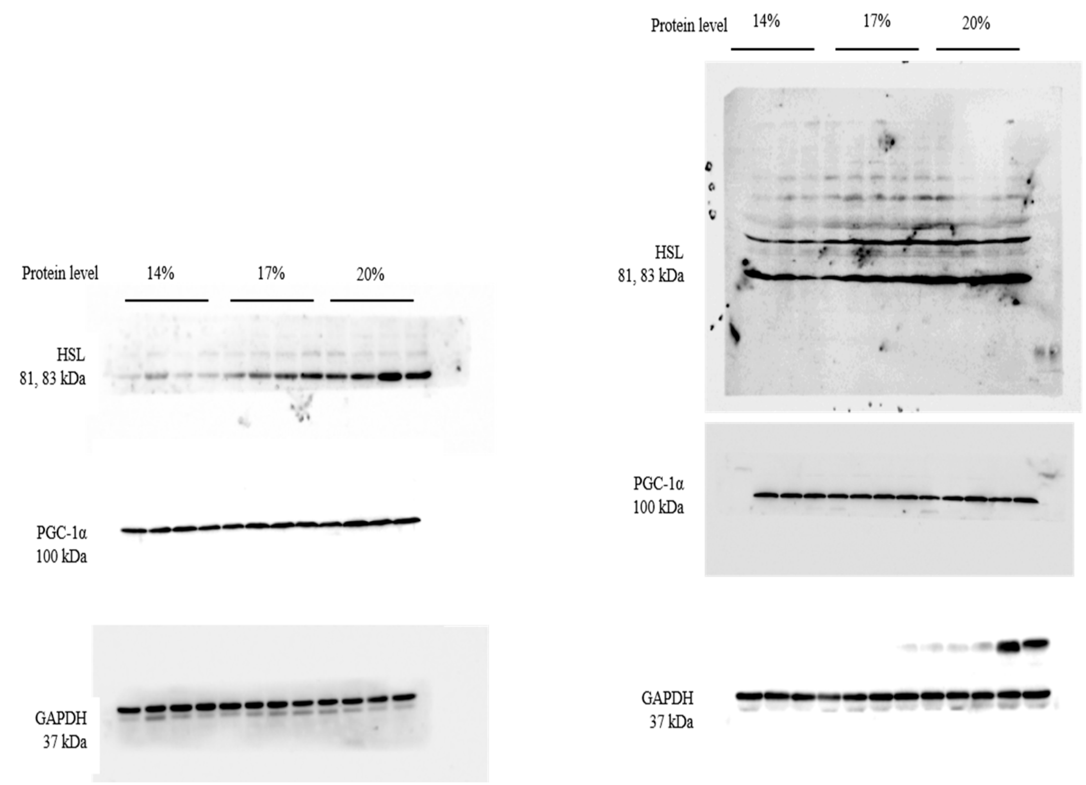

Supplemental Figure S2. Immunoblot analysis of Figure 3 AB

Online Supporting Material

Supplemental Table S1. Primers used in supplemental Figure 1 for real-time PCR

genes	accession no.	primers
ApoB	KP641122.1	F: 5'-GAGCAGTATTCAGCCAGCAC -3'<br>R: 5'-CCGACCATCATTGCCTTCTG -3'
ChREBP	XM_013995540.2	F: 5'-GTATGTGGAGCGGAGGAAGA -3'<br>R: 5'- CGCCACTTGTGGTATTCTCG -3'
CPT1B	NM_001007191.1	F: 5'- CCCAATTCCAGATCCGCATG-3'<br>R: 5'-TGAGAACTTGCTGGAGACGT-3'
HMGCR	NM_001122988.1	F: 5'- CTCAGCTCCAACTCACAGGA -3'<br>R: 5'- ACAAGAGCATCGAGGGTGAA -3'
Leptin	NM_213840.1	F: 5'- GCTCCATCCTGTCCTGAGTT-3'<br>R: 5'-AAGGCAGACTGGTGAGGATC -3'
MTTP	NM_214185.1	F: 5'-AGCCACCCCAAATCATCAGA -3'<br>R: 5'- GGTGGTCCCAGAGCTTAACT -3'
